# Examining the role of insulin-like growth factor 1 in the pathophysiology of carpal tunnel syndrome: a Mendelian randomization study

**DOI:** 10.1093/ije/dyag173

**Published:** 2026-08-27

**Authors:** Alex J Wood, Catherine E Lovegrove, Dominic Furniss, Akira Wiberg

**Affiliations:** Nuffield Department of Orthopaedics, Rheumatology and Musculoskeletal Sciences, University of Oxford, Oxford, United Kingdom; Nuffield Department of Surgical Sciences, University of Oxford, Oxford, United Kingdom; Department of Urology, Oxford University Hospitals NHS Foundation Trust, Oxford, United Kingdom; College of Medical, Veterinary and Life Sciences, University of Glasgow, Glasgow, United Kingdom; Nuffield Department of Orthopaedics, Rheumatology and Musculoskeletal Sciences, University of Oxford, Oxford, United Kingdom; Department of Plastic Surgery, Oxford University Hospitals Trust, Oxford, United Kingdom; Nuffield Department of Orthopaedics, Rheumatology and Musculoskeletal Sciences, University of Oxford, Oxford, United Kingdom; Department of Plastic Surgery, Oxford University Hospitals Trust, Oxford, United Kingdom

**Keywords:** carpal tunnel syndrome, Mendelian randomization, insulin-like growth factor 1

## Abstract

**Background:**

Carpal tunnel syndrome (CTS) is a common and disabling condition of the hand, affecting up to 10% of the population, caused by compression of the median nerve at the wrist. Conventional observational epidemiological studies have demonstrated associations between CTS and elevated plasma insulin-like growth factor 1 (IGF-1), as well as several metabolic factors, such as obesity and type 2 diabetes (T2DM).

**Methods:**

Here, we use two-sample univariable and multivariable Mendelian randomization to investigate causality in the relationships between IGF-1, metabolic factors related to IGF-1, and CTS.

**Results:**

We replicate the result that increases in measures of obesity (body mass index (BMI), waist-hip ratio (WHR), and waist circumference (WC)) are causal for CTS, and show that the effect of elevated childhood BMI on increasing risk of CTS is mediated mostly by elevated adult BMI. Furthermore, we demonstrate that T2DM and raised fasting plasma glucose are also causal for CTS. Finally, we show that increased IGF-1 signalling is also causally related to CTS risk, and the effect is likely mediated by its effects on both BMI and T2DM. The IGF-1 pathway merits further investigation as a potential therapeutic target for CTS.

**Conclusion:**

We provide genetic epidemiological evidence to support obesity, T2DM, and elevated plasma IGF-1 concentrations playing a causal role in increasing the risk of CTS. These findings suggest that pharmacological management of the metabolic risk factors may also decrease the risk of CTS, with the IGF-1 pathway as a potential therapeutic target.

Key Messages

- *Research question*: Using MR, this paper aims to identify whether elevated plasma IGF-1 and other associated metabolic factors may play a causal role in carpal tunnel syndrome pathophysiology, based on existing observational evidence.
- *Key findings*: This paper identifies that liability to obesity, type 2 diabetes, and elevated plasma IGF-1 concentrations may play an interlinked, causal role in increasing the risk of carpal tunnel syndrome.
- *Importance*: These modifiable risk factors could be targeted in future to improve carpal tunnel syndrome management.

## Introduction

Carpal tunnel syndrome (CTS) affects up to 10% of the population [1] and is caused by compression of the median nerve at the wrist. Patients experience disabling pain and paraesthesia in the median nerve distribution, and thumb weakness with detrimental effects on hand function, ability to work, and quality of life [2]. CTS is treated by carpal tunnel decompression surgery, the most common hand operation worldwide, which is costly to health services [3]. Up to 25% of patients do not improve or experience symptom recurrence following surgery [4]. CTS thus exacts a considerable socioeconomic burden [5].

The risk of CTS is affected by environmental and genetic factors. Heritability is estimated at 46% [6]. Large genome-wide association studies (GWAS) implicate the *DIRC3* variant, rs62175241, in CTS [7–9]. The T allele is protective, and is postulated to antagonize the insulin-like growth factor 1 (IGF-1) signalling pathway [8]. This single nucleotide polymorphism (SNP) is proposed to increase IGF Binding Protein 5 (IGFBP5) levels, a binding protein regulating IGF-1 signalling independently of IGF-1 receptor (IGF-1R) expression. IGFBP5 is bound to acid-labile subunit glycoprotein in the circulation and sequesters IGF-1 away from the IGF-1R [10], reducing IGF-1 signalling. Increased levels of IGFBP5 are identified in tenosynovium samples from patients with the protective SNP [8].

Observational evidence also implicates elevated IGF-1 in CTS pathophysiology. Epidemiological studies demonstrate associations between CTS and elevated plasma IGF-1 [8, 11]. Acromegaly, excess growth hormone (GH) which stimulates IGF-1 production, is a risk factor for CTS [12]; exogenous GH increases plasma IGF-1 and induces CTS which is reversed on withdrawing treatment [13].

IGF-1 plays a complex, time-dependent role in growth and development. IGF-1 is involved in skeletal muscle growth and maintenance [14], and regulates long bone growth at the growth plate [15]. Taller children have higher plasma IGF-1 [16], and children with lower birth weights also have higher plasma IGF-1, possibly related to re-programming of the axis to promote faster postnatal growth [17]. Plasma IGF-1 correlates with height increase in children taking supplemental GH for GH deficiency [18]. A recent Mendelian randomization (MR) study indicates that elevated IGF-1 leads to taller adult height [16]; observational data from UK Biobank shows that height is protective for CTS [7].

In adult males, plasma IGF-1 is inversely associated with BMI [19]; in adult females, it is inversely associated with abdominal sagittal diameter and visceral adipose tissue, perhaps reflecting intra-abdominal fat mass [20]. Transgenic mouse models implicate IGF-1 signalling in adipocyte lipid turnover [21]. Obesity (BMI > 30kgm^−2^) is reported as a risk factor for CTS [22], while bariatric surgery is transiently protective [23]. Waist-to-hip ratio (WHR), an alternative measure of obesity, may better reflect increased central obesity as opposed to generalized obesity; it is more directly associated with mortality and may be a better measure of metabolic health [24]. Waist circumference (WC), another measure of central adiposity, is associated with metabolic syndrome and increased all-cause mortality [25].

Higher childhood BMI is associated with elevated plasma [26] and urinary [27] IGF-1; overall IGF-1 is higher in female children [26]. Observational evidence suggests that childhood obesity may alter the risk of adult-onset diseases including breast cancer [28], coronary artery disease [29], and type 2 diabetes (T2DM) [30] independently of adult BMI. The effects of childhood obesity on risk of CTS have not been explored.

Altered IGF-1 signalling is also present in T2DM. High portal vein insulin levels reduce the threshold of GH signalling required for hepatic IGF-1 production, resulting in higher plasma IGF-1 within the normal range [31]. Many studies report increased incidence of CTS in patients with diabetes [32]. Both type 1 diabetes (T1DM) (pooled adjusted odds ratio (OR) 3.21 (95% confidence interval (CI) 1.24–8.28)) and T2DM (pooled adjusted OR 2.33 (95% CI 1.44–3.79)) were associated with CTS in a 25-study meta-analysis [33].

**Figure 1:**
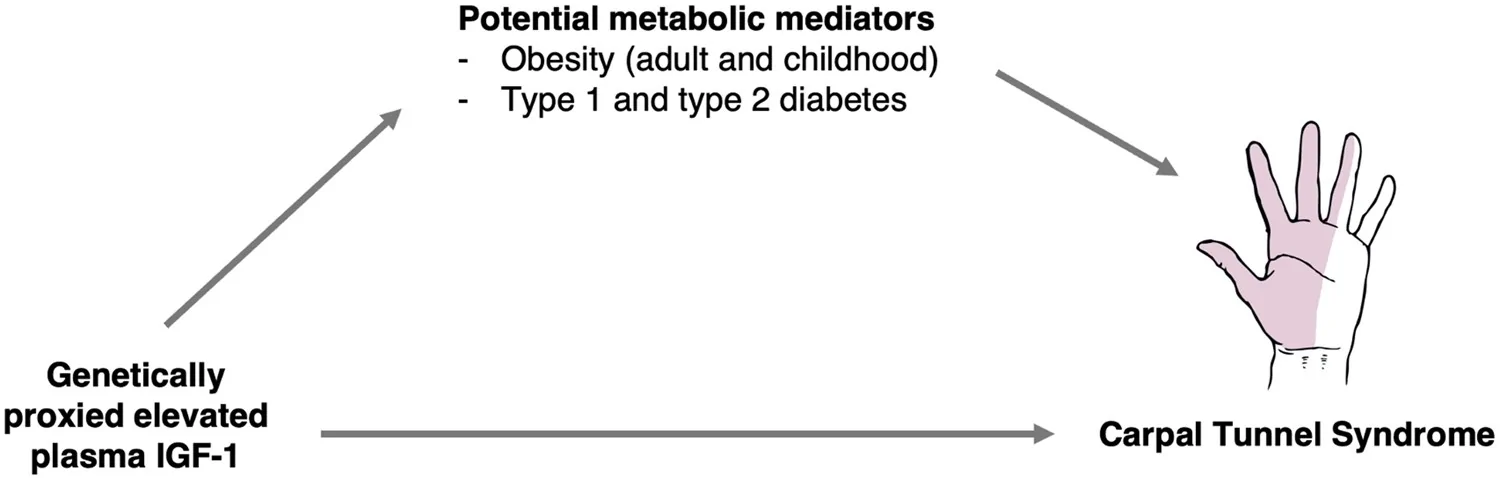
Flowchart indicating the analysis undertaken in this paper.

Our understanding of the role of IGF-1 in CTS aetiology is founded largely on observational studies. Observational evidence may be limited by residual confounding and reverse causation [34]. Conversely, MR, a subtype of instrumental variable analysis, is a genetic epidemiological approach utilizing SNPs associated with a phenotype of interest (exposure) to examine causal effects on another phenotype (outcome) [35]. MR CTS studies identify height as a protective factor [7], while obesity and hyperglycaemia increase CTS risk [36, 37]. These factors may be related to IGF-1 in CTS pathophysiology; we sought to use two-sample univariable and multivariable MR and mediation MR to disentangle the causal relationships between plasma IGF-1, relevant metabolic and anthropometric factors, and CTS, leveraging data from the most recent publicly available GWAS for CTS [9] (Fig. 1).

## Materials and methods

This study is reported in accordance with STROBE-MR guidelines (Supplementary Table S1) [38, 39].

### Study populations

For the outcome variable (CTS), we leveraged summary statistics from the most recent European GWAS meta-analysis of CTS [9]. This included 48 843 CTS cases (Iceland 8122; UK 19 849; Denmark 9664; Finland 11 208) and 1 190 837 controls (Iceland 318 161; UK 411 179; Denmark 266 450; Finland 195 047).

Exposure phenotype instrumental variables (IVs) were identified through literature searches for published GWAS (Table 1, Supplementary Table S2). GWAS-significant (*P* < 5 × 10^−8^) SNPs were selected and independent (*r*^2^ 0.001) SNPs ascertained using the clump_data() function from the TwoSampleMR package (v0.6.6) using a European population linkage disequilibrium (LD) reference panel. HbA1c is influenced by multiple genetic mechanisms [36], thus, only SNPs affecting HbA1c via glycaemic pathways were selected; SNPs acting via non-glycaemic pathways were used as a negative control.

**Table 1:** Summary of the GWAS datasets from which instrumental variables were drawn.

Exposure	Units	Population	Sample size	Number of independent IVs	**Reference**
Plasma IGF-1	SD, nmol/l	European	318 749	293	Neale lab [40]
Height	SD, cm	European	1 177 601	752	Yengo *et al.*, 2022 [41]
Whole body fat free mass	SD, kg	European	454 850	496	Elsworth, 2018 [42]
BMI	SD, kg/m2	European	694 649	281	Pulit *et al.*, 2019 [43]
WHR	SD, unitless	European	694 649	185	Pulit *et al.*, 2019 [43]
WC	SD, cm	European	142 762	37	Shungin *et al.*, 2015 [44]
Childhood BMI	SD, kg/m2	European	39 620	15	Vogelezang *et al.*, 2020 [45]
Type 1 diabetes mellitus	N/A	European	24 840	36	Forgetta *et al.*, 2021 [46]
Type 2 diabetes mellitus	N/A	European	933 970	166	Mahajan *et al.*, 2022 [47]
Fasting plasma glucose	SD, mmol/l	European	314 916	90	Sakaue *et al.*, 2021 [48]
HbA1c (glycaemic)	SD, %	European	151 013	7	Chen *et al.*, 2021 [49]
HbA1c (non-glycaemic)	SD. %	European	151 013	24	Chen *et al.*, 2021 [49]
Fasting plasma insulin	SD, pmol/l	European	151 013	34	Chen *et al.*, 2021 [49]
Triglycerides	SD, mmol/l	European	864 240	213	Graham *et al.*, 2021 [50]
HDL	SD, mmol/l	European	888 227	224	Graham *et al.*, 2021 [50]
LDL	SD, mmol/l	European	842 660	213	Graham *et al.*, 2021 [50]
Total cholesterol	SD, log()	European	930 672	253	Graham *et al.*, 2021 [50]
Pulse pressure	SD, mmHg	European	757 601	29	Evangelou *et al.*, 2018 [51]
Diastolic blood pressure	SD, mmHg	European	757 601	35	Evangelou *et al.*, 2018 [51]
Systolic blood pressure	SD, mmHg	European	757 601	17	Evangelou *et al.*, 2018 [51]

IVs, instrumental variables; IGF-1, insulin-like growth factor 1; BMI, body mass index; WHR, waist–hip ratio; WC, waist circumference; HbA1c, glycated haemoglobin; HDL, high-density lipoprotein; LDL, low-density lipoprotein; SD, standard deviation.

### Mendelian randomization

Inverse variance weighted (IVW), MR-Egger, weighted median (Supplementary Table S4), and multivariable MR analyses were performed using MendelianRandomisation (v0.10.0) and TwoSampleMR (v0.6.29) in R (v4.5.2). MR-PRESSO (Supplementary Table S5) was performed using MRPRESSO (v1.0).

To assess the plausibility of the core IV assumptions (relevance, independence, and exclusion restriction [35]), mean F statistics and total *R*^2^ across exposure IVs were calculated using formulae previously reported (Supplementary Tables S2 and S3) [52]. Cochran’s *Q* test was used to determine heterogeneity in the derived MR estimate.

Harmonization and Steiger filtering were performed for exposure and outcome data where three or more significant, associated, and independent SNPs were available, and allele frequency was used for palindromic SNPs to infer positive strand alleles. SNPs were omitted if harmonization was not possible and positive strand alleles remained ambiguous.

**Figure 2:**
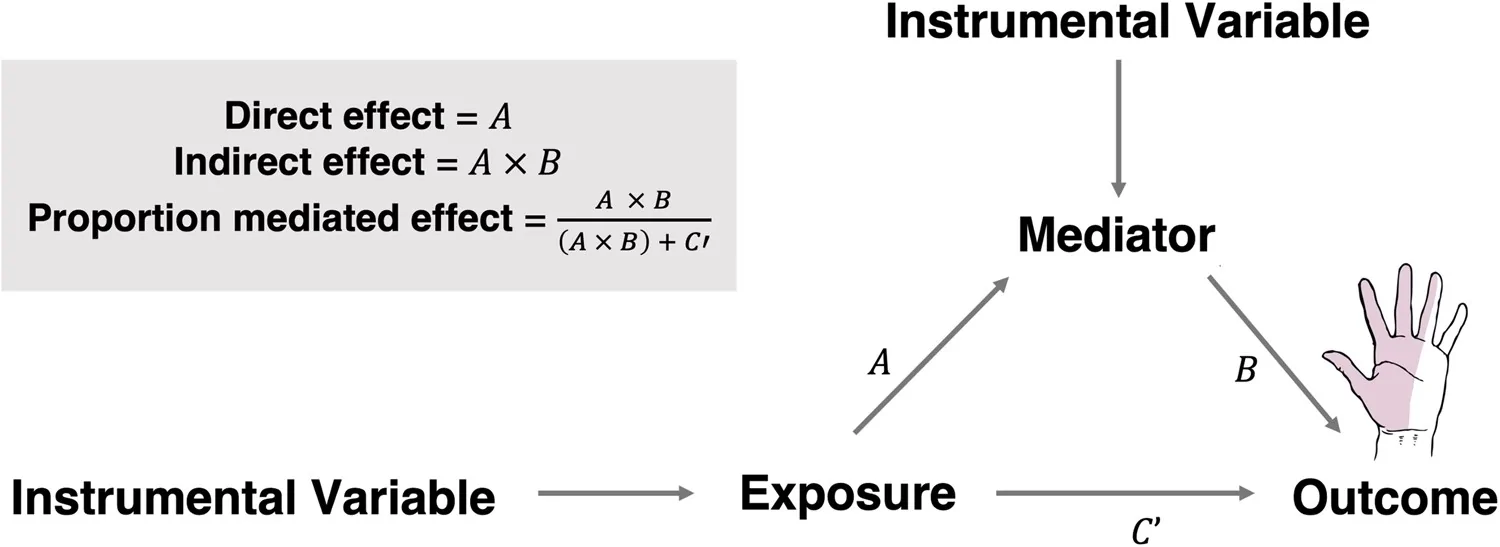
Directed acyclic graph (DAG) and relevant equations indicating the methods used for mediation MR analysis.

Where MR-Egger intercept was non-zero with *P* < 0.05, suggesting potential horizontal pleiotropy, MR-Egger results were used [53]; otherwise, IVW results were used as estimate of best fit. Leave-one-out analysis omitting each IV was undertaken if significant effects were identified from univariable MR (Supplementary Table S6). Multivariable MR was performed to examine the effect of IVs on possible confounding variables with a known biological association. Mediation analysis was performed as previously reported (Fig. 2) [52] to examine direct and indirect effects of factors potentially acting on the same causal pathway, and 95% CIs were calculated via the bootstrapping method.

*P*-values were adjusted using the Benjamini–Hochberg false discovery rate method [54], controlled at 5%, to account for multiple testing involving closely related traits.

## Results

### IGF-1 and CTS

**Figure 3:**
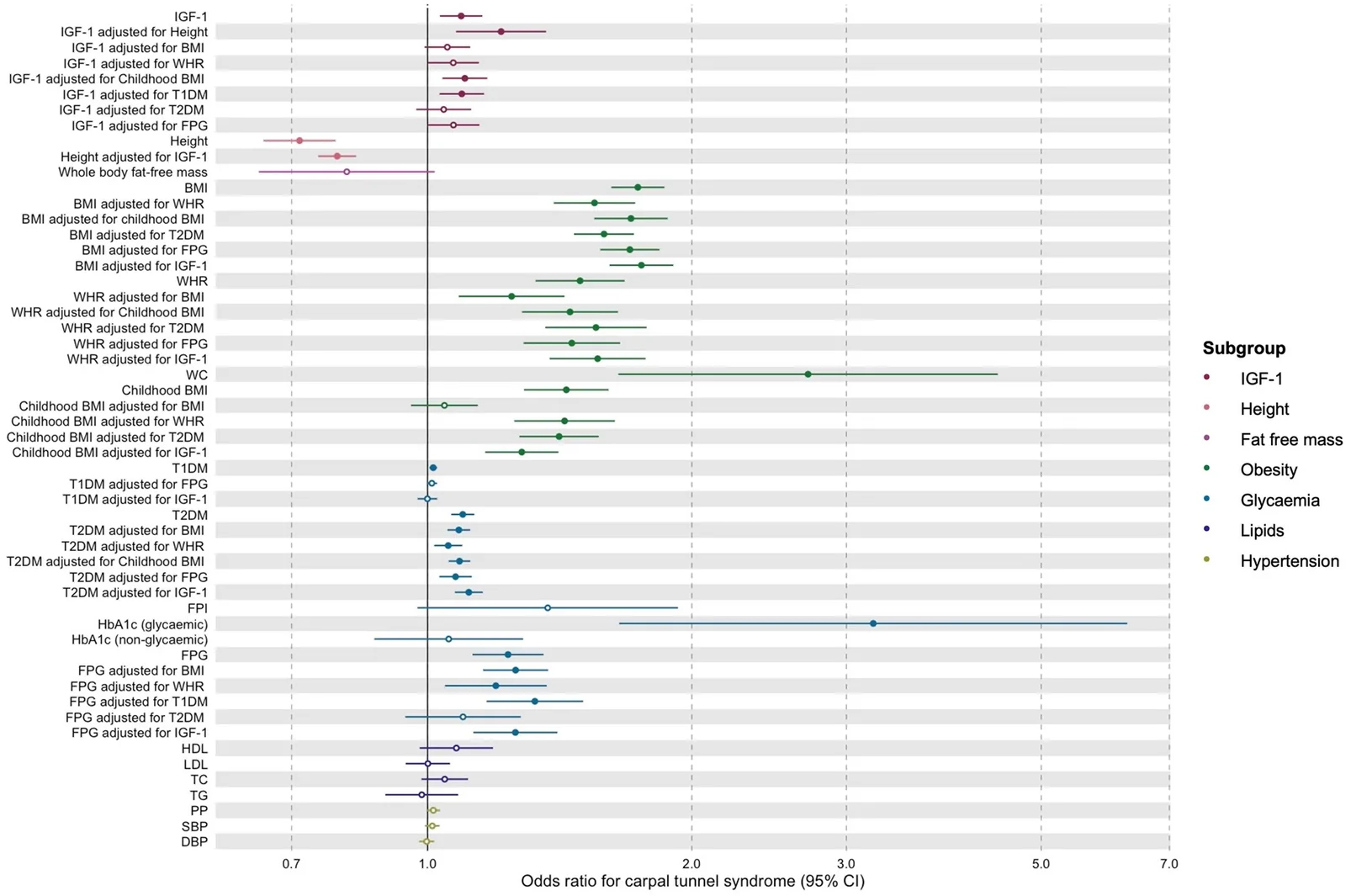
Forest plot of odds ratios and 95% confidence intervals (95% CI) of carpal tunnel syndrome per 1-standard deviation higher genetically instrumented exposure variable from univariable and multivariable Mendelian randomization analysis. IGF-1, insulin-like growth factor 1; BMI, body mass index; WHR, waist–hip ratio; T1DM, type 1 diabetes mellitus; T2DM, type 2 diabetes mellitus; FPG, fasting plasma glucose; FPI, fasting plasma insulin; HbA1c, glycated haemoglobin; HDL, high-density lipoprotein; LDL, low-density lipoprotein; TC, total cholesterol; TG, triglycerides; PP, pulse pressure; SBP, systolic blood pressure; DBP, diastolic blood pressure.

Using univariable MR, genetically instrumented higher plasma IGF-1 increased the odds of CTS (odds ratio (OR) 1.09 [95% confidence interval (CI) 1.03–1.15, *P* = 3.89 × 10^−3^]) (Table 2, Fig. 3, Supplementary Fig. S1). Given the relationships between IGF-1, growth, adiposity, and glycaemic traits, we examined whether the association between IGF-1 and CTS persisted after adjustment for potential confounding metabolic factors using multivariable MR. Specifically, we examined height, BMI, WHR, childhood BMI, T1DM, T2DM, and fasting plasma glucose (FPG).

**Table 2:** Univariable MR analyses for exposures on the risk of carpal tunnel syndrome.

Exposure	Number of SNPs	Inverse variance weighted				MR Egger					
		Estimate		Heterogeneity		Estimate		Intercept		Heterogeneity	
		Odds ratio (95% CI)	*P*-value (adjusted)	*Q*	*P*-value	Odds ratio (95% CI)	*P*-value (adjusted)	Estimate (SE)	*P*-value	*Q*	*P*-value
Plasma IGF-1	293	1.09 (1.03–1.15)	**3.89 × 10** ^−^^**3**^	798.12	1.35 × 10^−48^	1.09 (0.98–1.21)	0.297	2.06 × 10^−4^ (1.55 × 10^−3^)	0.894	798.07	8.27 × 10^−49^
Height	752	0.80 (0.76–0.83)	2.95 × 10^−21^	2218.16	1.12 × 10^−144^	0.71 (0.65–0.79)	**1.69 × 10** ^−^^**10**^	2.61 × 10^−3^ (1.03 × 10^−3^)	0.012	2199.5	3.13 × 10^−142^
Whole body fat-free mass	496	1.11 (1.00–1.22)	0.072	1738.79	9.20 × 10^−138^	0.81 (0.64–1.02)	0.207	4.70 × 10^−3^ (1.60 × 10^−3^)	3.48 × 10^−3^	1708.96	2.09 × 10^−133^
BMI	281	1.74 (1.62–1.86)	**7.89 × 10** ^−^^**53**^	508.74	1.76 × 10^−15^	1.74 (1.47–2.06)	4.83 × 10^−9^	−7.34 × 10^−5^ (1.52 × 10^−3^)	0.962	508.73	1.30 × 10^−15^
WHR	185	1.49 (1.33–1.68)	**1.27 × 10** ^−^^**10**^	556.85	3.89 × 10^−39^	1.31 (0.9–1.91)	0.321	2.27 × 10^−3^ (3.23 × 10^−3^)	0.483	555.35	3.69 × 10^−39^
WC	37	1.48 (1.21–1.82)	3.48 × 10^−4^	217.95	6.74 × 10^−28^	2.71 (1.65–4.46)	**1.91 × 10** ^−^^**3**^	−0.020 (7.64 × 10^−3^)	0.015	183.39	5.11 × 10^−22^
Childhood BMI	15	1.44 (1.29–1.61)	**6.72 × 10** ^−^^**10**^	45.45	3.45 × 10^−5^	1.25 (0.78–2.01)	0.598	9.87 × 10^−3^ (0.017)	0.561	44.24	2.80 × 10^−5^
Type 1 diabetes mellitus	36	1.01 (1.00–1.03)	**6.81 × 10** ^−^^**3**^	97.42	8.55 × 10^−8^	1.02 (1.01–1.04)	0.023	−4.39 × 10^−3^ (3.61 × 10^−3^)	0.232	93.35	1.95 × 10^−7^
Type 2 diabetes mellitus	166	1.10 (1.06–1.13)	**1.10 × 10** ^−^^**8**^	421.55	2.21 × 10^−24^	1.14 (1.06–1.21)	1.59 × 10^−3^	−2.58 × 10^−3^ (2.24 × 10^−3^)	0.252	418.18	3.87 × 10^−24^
Fasting plasma glucose	90	1.23 (1.13–1.36)	**2.80 × 10** ^−^^**5**^	251.82	1.78 × 10^−17^	1.18 (1.02–1.36)	0.095	1.93 × 10^−3^ (2.40 × 10^−3^)	0.423	249.98	1.92 × 10^−17^
HbA1c (glycaemic)	7	3.22 (1.65–6.27)	**1.30 × 10** ^−^^**3**^	23.07	7.73 × 10^−4^	1.58 (0.38–6.65)	0.801	0.015 (0.014)	0.325	18.64	2.24 × 10^−4^
HbA1c (non-glycaemic)	24	1.06 (0.87–1.28)	0.642	31.97	0.101	0.99 (0.72–1.35)	0.993	1.69 × 10^−3^ (3.11 × 10^−3^)	0.592	31.55	0.0854
Fasting plasma insulin	34	1.37 (0.97–1.93)	0.101	148.18	1.54 × 10^−16^	1.67 (0.6–4.67)	0.598	−3.65 × 10^−3^ (9.00 × 10^−3^)	0.688	147.43	9.56 × 10^−17^
HDL	224	0.93 (0.88–0.99)	0.035	658.21	1.55 × 10^−44^	1.08 (0.98–1.19)	0.297	−5.93 × 10^−3^ (1.57 × 10^−3^)	2.05 × 10^−4^	618.48	4.16 × 10^−39^
LDL	213	1.00 (0.94–1.06)	0.980	536.81	4.33 × 10^−30^	1.00 (0.92–1.09)	0.993	5.10 × 10^−5^ (1.44 × 10^−3^)	0.972	536.8	2.71 × 10^−30^
Total cholesterol	253	1.05 (0.98–1.11)	0.186	738.81	2.58 × 10^−49^	1.01 (0.91–1.11)	0.993	1.45 × 10^−3^ (1.52 × 10^−3^)	0.339	736.11	3.66 × 10^−49^
Triglycerides	213	1.16 (1.08–1.23)	2.80 × 10^−5^	632.71	1.85 × 10^−43^	0.98 (0.90–1.08)	0.944	6.46 × 10^−3^ (1.48 × 10^−3^)	2.04 × 10^−5^	580.43	3.06 × 10^−36^
Pulse pressure	29	1.02 (1.00–1.03)	0.121	77.25	1.70 × 10^−6^	1.03 (0.97–1.09)	0.598	−3.35 × 10^−3^ (8.49 × 10^−3^)	0.696	76.81	1.14 × 10^−6^
Systolic blood pressure	17	1.01 (0.99–1.03)	0.239	49.1	3.19 × 10^−5^	1.00 (0.92–1.10)	0.993	3.73 × 10^−3^ (0.016)	0.820	48.92	1.80 × 10^−5^
Diastolic blood pressure	35	1.00 (0.98–1.02)	0.857	83	5.59 × 10^−6^	1.02 (0.94–1.10)	0.912	−4.15 × 10^−3^ (8.56 × 10^−3^)	0.631	82.41	4.14 × 10^−6^

The primary estimate of effect was taken from the inverse variance weighted method; where there was evidence of horizontal pleiotropy (intercept *P* < 0.05) the MR-Egger estimate was interpreted as the most relevant estimate. Significant *P*-values (corrected using Benjamini–Hochberg correction) are highlighted in bold.

Number of SNPs, number of single-nucleotide polymorphisms used as instrumental variables; CI, confidence interval; *Q*, *Q* statistic; SE, standard error; IGF-1, insulin-like growth factor 1; BMI, body mass index; WHR, waist–hip ratio; WC, waist circumference; HbA1c, glycated haemoglobin; HDL, high-density lipoprotein; LDL, low-density lipoprotein.

In multivariable MR, higher IGF-1 remained causally related to CTS when adjusted for height, childhood BMI, and T1DM. The effect of plasma IGF-1 on CTS attenuated and was no longer significant after adjustment for BMI, WHR, T2DM, and FPG. These findings suggest that the effect of IGF-1 on CTS risk may be partially acting via pathways related to adult adiposity and glycaemic dysregulation.

### Height, muscle mass, and CTS

Height was examined as a biologically related trait to IGF-1 and as a potential confounder of CTS risk. Genetically instrumented taller height was protective for CTS (Table 2), and attenuated when adjusted for IGF-1 (Table 3). Fat-free muscle mass had no effect on odds of CTS (Table 2).

**Table 3:** Multivariable MR analyses for exposures on the risk of carpal tunnel syndrome.

Exposure	Adjusted for	Number of SNPs	Inverse variance weighted				MR Egger					
			Estimate		Heterogeneity		Estimate		Intercept		Heterogeneity	
			Odds ratio	*P*-value	*Q*	*P*-value	Odds ratio	*P*-value	Estimate	*P*-value	*Q*	*P*-value
IGF-1	Height	672	1.21 (1.08–1.36)	**2.11 × 10** ^−^^**3**^	1971.15	3.19 × 10^−128^	1.12 (0.96–1.31)	0.176	1.05 × 10^−3^ (6.93 × 10^−4^)	0.130	1964.42	1.72 × 10^−127^
IGF-1	BMI	365	1.05 (0.99–1.12)	0.106	753.88	1.46 × 10^−29^	1.07 (0.98–1.16)	0.160	−3.94 × 10^−4^ (8.56 × 10^−4^)	0.646	753.45	1.13 × 10^−29^
IGF-1	WHR	314	1.07 (1.00–1.14)	0.0613	883.82	4.11 × 10^−56^	1.06 (0.96–1.17)	0.254	2.43 × 10^−4^ (1.14 × 10^−3^)	0.832	883.70	2.53 × 10^−56^
IGF-1	Childhood BMI	279	1.10 (1.04–1.17)	**1.85 × 10** ^−^^**3**^	783.44	6.75 × 10^−50^	1.08 (0.97–1.21)	0.182	5.90 × 10^−4^ (1.59 × 10^−3^)	0.710	783.06	4.53 × 10^−50^
IGF-1	T1DM	283	1.09 (1.03–1.16)	**3.69 × 10** ^−^^**3**^	772.63	1.75 × 10^−47^	1.09 (1.03–1.16)	4.18 × 10^−3^	−3.59 × 10^−4^ (1.00 × 10^−3^)	0.720	772.28	1.17 × 10^−47^
IGF-1	T2DM	305	1.04 (0.97–1.12)	0.266	879.31	1.53 × 10^−57^	1.05 (0.94–1.17)	0.455	−6.46 × 10^−5^ (1.23 × 10^−3^)	0.958	879.31	8.93 × 10^−58^
IGF-1	FPG	286	1.07 (1.00–1.15)	0.0613	856.06	1.10 × 10^−58^	1.09 (0.97–1.24)	0.188	−6.35 × 10^−4^ (1.58 × 10^−3^)	0.687	855.58	7.41 × 10^−59^
Height	IGF-1	672	0.79 (0.75–0.83)	**7.44 × 10** ^−^^**20**^	1971.15	3.19 × 10^−128^	0.79 (0.75–0.83)	1.22 × 10^−19^	1.05 × 10^−3^ (6.93 × 10^−4^)	0.130	1964.42	1.72 × 10^−127^
BMI	IGF-1	365	1.75 (1.61–1.90)	**3.32 × 10** ^−^^**38**^	753.88	1.46 × 10^−29^	1.74 (1.60–1.90)	3.49 × 10^−34^	−3.94 × 10^−4^ (8.56 × 10^−4^)	0.646	753.45	1.13 × 10^−29^
BMI	WHR	334	1.55 (1.39–1.72)	**5.66 × 10** ^−^^**15**^	731.50	4.04 × 10^−32^	1.66 (1.40–1.96)	1.99 × 10^−8^	−1.24 × 10^−3^ (1.26 × 10^−3^)	0.324	729.36	4.89 × 10^−32^
BMI	Childhood BMI	290	1.70 (1.55–1.88)	**1.72 × 10** ^−^^**26**^	517.98	2.40 × 10^−15^	1.71 (1.55–1.88)	3.66 × 10^−26^	−3.12 × 10^−4^ (8.63 × 10^−4^)	0.717	517.75	1.87 × 10^−15^
BMI	T2DM	339	1.59 (1.47–1.72)	**1.01 × 10** ^−^^**29**^	656.36	8.71 × 10^−23^	1.65 (1.49–1.82)	2.27 × 10^−22^	−1.37 × 10^−3^ (1.12 × 10^−3^)	0.220	653.43	1.28 × 10^−22^
BMI	FPG	315	1.70 (1.57–1.84)	**2.79 × 10** ^−^^**39**^	619.88	2.05 × 10^−22^	1.72 (1.58–1.87)	2.34 × 10^−33^	−4.34 × 10^−4^ (7.94 × 10^−4^)	0.584	619.29	1.68 × 10^−22^
WHR	IGF-1	314	1.56 (1.38–1.77)	**1.88 × 10** ^−^^**11**^	883.82	4.11 × 10^−56^	1.57 (1.38–1.79)	7.95 × 10^−11^	2.43 × 10^−4^ (1.14 × 10^−3^)	0.832	883.70	2.53 × 10^−56^
WHR	BMI	334	1.25 (1.09–1.43)	**2.74 × 10** ^−^^**3**^	731.50	4.04 × 10^−32^	1.25 (1.09–1.44)	2.79 × 10^−3^	−1.24 × 10^−3^ (1.26 × 10^−3^)	0.324	729.36	4.89 × 10^−32^
WHR	Childhood BMI	198	1.43 (1.26–1.63)	7.70 × 10^−8^	629.04	7.15 × 10^−47^	1.45 (1.28–1.65)	**1.99 × 10** ^−^^**8**^	−3.20 × 10^−3^ (1.31 × 10^−3^)	0.0149	610.30	2.54 × 10^−44^
WHR	T2DM	270	1.56 (1.36–1.78)	**2.88 × 10** ^−^^**10**^	779.45	2.11 × 10^−51^	1.59 (1.36–1.85)	1.49 × 10^−8^	−9.29 × 10^−4^ (1.53 × 10^−3^)	0.543	777.33	2.48 × 10^−51^
WHR	FPG	231	1.46 (1.29–1.66)	**1.46 × 10** ^−^^**8**^	730.54	1.01 × 10^−53^	1.49 (1.30–1.70)	1.99 × 10^−8^	−9.51 × 10^−4^ (1.19 × 10^−3^)	0.424	728.51	1.14 × 10^−53^
Childhood BMI	IGF-1	279	1.28 (1.16–1.41)	**1.03 × 10** ^−^^**6**^	783.44	6.75 × 10^−50^	1.28 (1.16–1.42)	1.16 × 10^−6^	5.90 × 10^−4^ (1.59 × 10^−3^)	0.710	783.06	4.53 × 10^−50^
Childhood BMI	BMI	290	1.05 (0.96–1.14)	0.332	517.98	2.40 × 10^−15^	1.06 (0.95–1.17)	0.331	−3.12 × 10^−4^ (8.63 × 10^−4^)	0.717	517.75	1.87 × 10^−15^
Childhood BMI	WHR	198	1.30 (1.17–1.44)	2.97 × 10^−6^	629.04	7.15 × 10^−47^	1.43 (1.26–1.63)	**2.24 × 10** ^−^^**7**^	−3.20 × 10^−3^ (1.31 × 10^−3^)	0.0149	610.30	2.54 × 10^−44^
Childhood BMI	T2DM	182	1.32 (1.22–1.43)	9.18 × 10^−11^	436.97	2.13 × 10^−23^	1.41 (1.27–1.57)	**4.17 × 10** ^−^^**10**^	−2.44 × 10^−3^ (1.21 × 10^−3^)	0.0448	427.36	2.36 × 10^−22^
T1DM	IGF-1	283	1.00 (0.97–1.03)	0.972	772.63	1.75 × 10^−47^	1.00 (0.97–1.03)	0.866	−3.59 × 10^−4^ (1.00 × 10^−3^)	0.720	772.28	1.17 × 10^−47^
T1DM	FPG	111	1.01 (1.00–1.03)	0.124	357.22	3.67 × 10^−28^	1.01 (1.00–1.03)	0.108	−1.32 × 10^−3^ (1.71 × 10^−3^)	0.439	355.25	4.02 × 10^−28^
T2DM	IGF-1	305	1.11 (1.07–1.16)	**2.06 × 10** ^−^^**8**^	879.31	1.53 × 10^−57^	1.11 (1.07–1.16)	2.18 × 10^−8^	−6.46 × 10^−5^ (1.23 × 10^−3^)	0.958	879.31	8.93 × 10^−58^
T2DM	BMI	339	1.09 (1.05–1.12)	**1.76 × 10** ^−^^**7**^	656.36	8.71 × 10^−23^	1.11 (1.06–1.15)	5.53 × 10^−6^	−1.37 × 10^−3^ (1.12 × 10^−3^)	0.220	653.43	1.28 × 10^−22^
T2DM	WHR	270	1.06 (1.02–1.10)	**5.49 × 10** ^−^^**3**^	779.45	2.11 × 10^−51^	1.07 (1.01–1.13)	0.0223	−9.29 × 10^−4^ (1.53 × 10^−3^)	0.543	777.33	2.48 × 10^−51^
T2DM	Childhood BMI	182	1.09 (1.06–1.12)	9.35 × 10^−9^	436.97	2.13 × 10^−23^	1.09 (1.06–1.12)	**2.21 × 10** ^−^^**8**^	−2.44 × 10^−3^ (1.21 × 10^−3^)	0.0448	427.36	2.36 × 10^−22^
T2DM	FPG	199	1.08 (1.03–1.12)	**1.23 × 10** ^−^^**3**^	535.45	4.42 × 10^−33^	1.04 (0.99–1.10)	0.165	2.99 × 10^−3^ (1.75 × 10^−3^)	0.087	527.57	3.27 × 10^−32^
FPG	IGF-1	286	1.26 (1.13–1.41)	**7.73 × 10** ^−^^**5**^	856.06	1.10 × 10^−58^	1.26 (1.13–1.40)	9.09 × 10^−5^	−6.35 × 10^−4^ (1.58 × 10^−3^)	0.687	855.58	7.41 × 10^−59^
FPG	BMI	315	1.26 (1.16–1.37)	**2.65 × 10** ^−^^**7**^	619.88	2.05 × 10^−22^	1.28 (1.16–1.41)	1.70 × 10^−6^	−4.34 × 10^−4^ (7.94 × 10^−4^)	0.584	619.29	1.68 × 10^−22^
FPG	WHR	231	1.20 (1.05–1.37)	**0.0112**	730.54	1.01 × 10^−53^	1.24 (1.06–1.46)	0.0136	−9.51 × 10^−4^ (1.19 × 10^−3^)	0.424	728.51	1.14 × 10^−53^
FPG	T1DM	111	1.33 (1.17–1.50)	**2.56 × 10** ^−^^**5**^	357.22	3.67 × 10^−28^	1.32 (1.16–1.50)	3.79 × 10^−5^	−1.32 × 10^−3^ (1.71 × 10^−3^)	0.439	355.25	4.02 × 10^−28^
FPG	T2DM	199	1.10 (0.94–1.28)	0.251	535.45	4.42 × 10^−33^	1.05 (0.89–1.23)	0.571	2.99 × 10^−3^ (1.75 × 10^−3^)	0.087	527.57	3.27 × 10^−32^

The primary estimate of effect was taken from the inverse variance weighted method; where there was evidence of horizontal pleiotropy (intercept *P* < 0.05) the MR-Egger estimate was interpreted as the most relevant estimate. Significant *P*-values (corrected using Benjamini–Hochberg correction) are highlighted in bold.

Number of SNPs, number of single-nucleotide polymorphisms used as instrumental variables; CI, confidence interval; *Q*, *Q* statistic; SE, standard error; IGF-1, insulin-like growth factor 1; BMI, body mass index; WHR, waist–hip ratio; WC, waist circumference; T1DM, type 1 diabetes mellitus; T2DM, type 2 diabetes mellitus; FPG, fasting plasma glucose.

### Obesity and CTS

All studied measures of adiposity (BMI, WHR, WC, childhood BMI) caused higher odds of CTS in univariable MR (Table 2). WHR was selected over WC for downstream analyses because it comprised a larger IV, providing a more precise effect estimate, and both are measures of central adiposity [25]. In multivariable MR, BMI and WHR remained significantly associated with increased odds of CTS after adjustment for metabolic factors that could confound this relationship (plasma IGF-1, T2DM, FPG, and childhood BMI). Both measures remained associated after mutual adjustment, allowing assessment of the independent contributions of generalized and central adiposity to CTS risk (Table 3). Childhood BMI remained significant following adjustment for WHR, plasma IGF-1, T2DM, and FPG, but was not when adjusted for adult BMI (Table 3). Thus, generalized and central adiposity independently increase CTS risk and childhood adiposity exerts effects largely through adult BMI.

### Glycaemic traits and CTS

**Figure 4:**
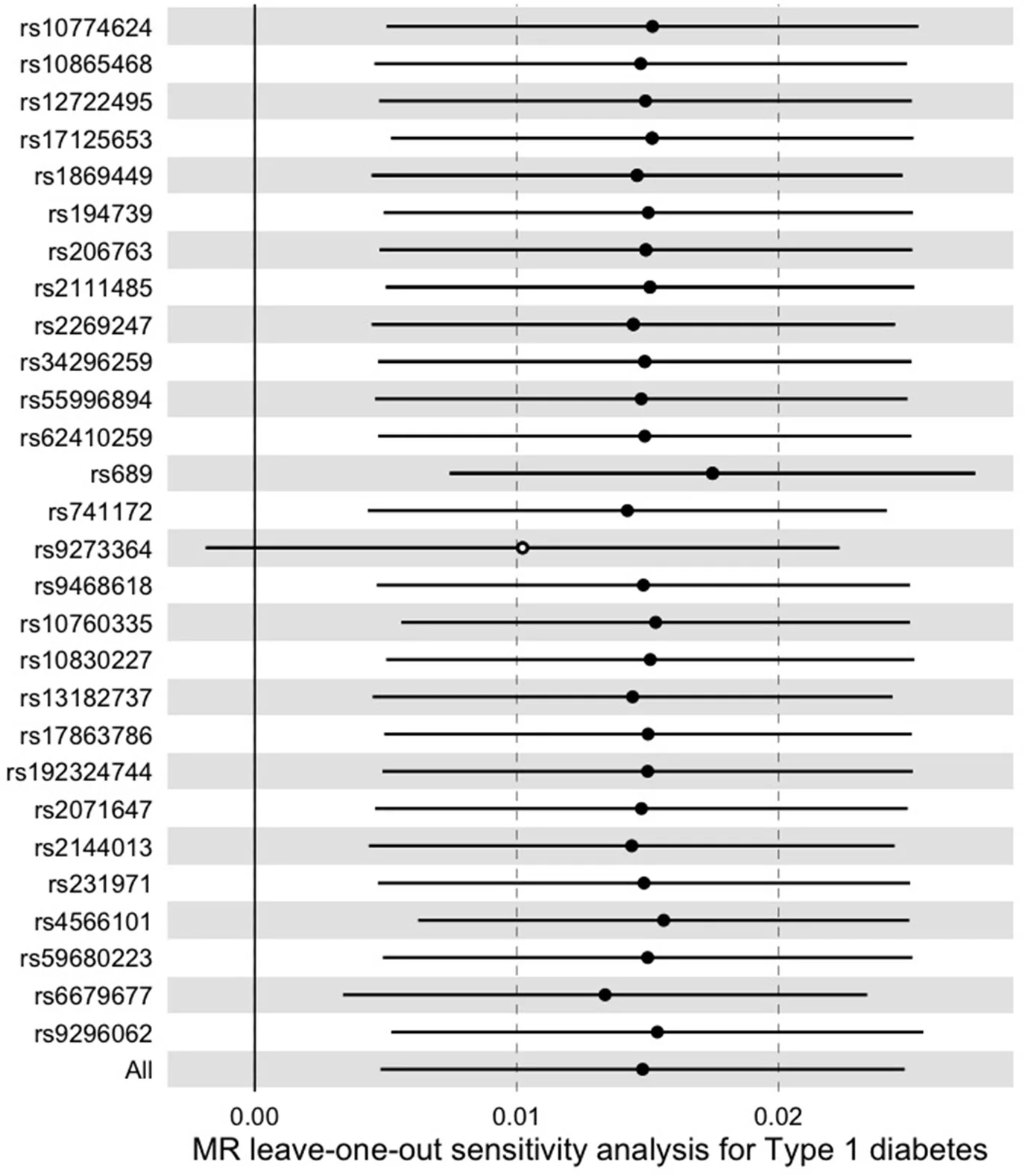
Forest plot showing the results of leave-one-out MR analysis for the exposure type 1 diabetes mellitus (T1DM).

T1DM, T2DM, FPG, and HbA1c all resulted in increased odds of CTS in univariable MR, while fasting plasma insulin did not have an effect (Table 2). FPG was selected over HbA1c for downstream analyses because it comprised a larger IV, providing a more precise effect estimate. T1DM no longer increased odds of CTS when adjusted for IGF-1 or FPG (Table 3). T2DM increased odds of CTS after adjusting for plasma IGF-1, BMI, WHR, childhood BMI, and FPG (Table 3). FPG remained significant when adjusted for plasma IGF-1, BMI, WHR, and T1DM, but attenuated when adjusted for T2DM (Table 3). Leave-one-out analysis for T1DM revealed that the association is driven by rs9273364 (Fig. 4).

### Dyslipidaemia, hypertension, and CTS

Univariable MR demonstrated no relationship between triglycerides, HDL, LDL, or total cholesterol and CTS (Table 2), nor pulse pressure, systolic blood pressure, or diastolic blood pressure and CTS (Table 2).

### Bidirectionality and mediation analysis

To untangle complex interactions between IGF-1 and metabolic factors, we performed bidirectional MR analyses to determine the directionality of these associations. Genetically instrumented higher plasma IGF-1 results in higher BMI, while genetically instrumented increases in adult and childhood BMI lower plasma IGF-1 (Table 4) suggesting a potential feedback loop or time-varying interaction between these phenotypes. Higher IGF-1 also increased risk of T2DM (Table 4). As adult BMI cannot affect childhood BMI, bidirectional MR was not undertaken.

**Table 4:** Bidirectional MR analyses.

Exposure	Outcome	Number of SNPs	Inverse variance weighted					MR Egger						
			Estimate			Heterogeneity		Estimate			Intercept		Heterogeneity	
			Beta (SE)	Odds ratio (95% CI)	*P*-value (adjusted)	*Q*	*P*-value	Beta (SE)	Odds ratio (95% CI)	*P*-value (adjusted)	Estimate (SE)	*P*-value	*Q*	*P*-value
BMI	IGF-1	292	−0.0973 (0.0186)		**1.01 × 10** ^−^^**6**^	1351.47	5.17 × 10^−136^	−0.100 (0.0454)		0.0844	5.63 × 10^−5^ (8.09 × 10^−4^)	0.945	1351.45	2.41 × 10^−136^
IGF-1	BMI	289	7.31 × 10^−3^ (0.0129)		0.570	2375.19	0	0.0788 (0.0236)		**1.68 × 10** ^−^^**3**^	−2.41 × 10^−3^ (6.74 × 10^−4^)	4.06 × 10^−4^	2273.73	1.79 × 10^−305^
Childhood BMI	IGF-1	16	−0.0791 (0.0333)		**0.0350**	159.46	3.16 × 10^−26^	0.0503 (0.137)		0.719	−9.28 × 10^−3^ (9.54 × 10^−3^)	0.347	149.36	9.67 × 10^−25^
IGF-1	Childhood BMI	258	−8.16 × 10^−4^ (0.0232)		0.972	328.26	1.74 × 10^−3^	0.0436 (0.0442)		0.324	−1.47 × 10^−3^ (1.24 × 10^−3^)	0.239	326.49	1.89 × 10^−3^
T2DM	IGF-1	82	7.71 × 10^−3^ (0.0115)		0.503	559.01	1.59 × 10^−72^	0.0190 (0.0221)		0.470	−9.43 × 10^−4^ (1.57 × 10^−3^)	0.549	556.49	1.77 × 10^−72^
IGF-1	T2DM	288	0.112 (0.0341)	1.12 (1.05–1.20)	**1.23 × 10** ^−^^**3**^	1267.72	4.03 × 10^−123^	0.188 (0.0637)	1.21 (1.07–1.37)	9.06 × 10^−3^	−2.57 × 10^−3^ (1.81 × 10^−3^)	0.158	1258.9	5.83 × 10^−122^
FPG	IGF-1	102	−0.0392 (0.0234)		0.140	675.35	8.82 × 10^−86^	−0.0551 (0.0371)		0.281	−6.59 × 10^−4^ (1.19 × 10^−3^)	0.581	673.29	8.19 × 10^−86^
IGF-1	FPG	330	0.0271 (9.34 × 10^−3^)		**5.91 × 10** ^−^^**3**^	1168.08	2.52 × 10^−94^	0.0534 (0.0187)		8.98 × 10^−3^	−8.52 × 10^−4^ (5.18 × 10^−4^)	0.101	1158.53	4.15 × 10^−93^
Childhood BMI	BMI	16	0.537 (0.0575)		**3.23 × 10** ^−^^**20**^	890.4	4.01 × 10^−180^	0.407 (0.245)		0.139	9.30 × 10^−3^ (0.0171)	0.594	871.91	4.50 × 10^−177^
BMI	FPG	296	0.140 (0.0101)		**3.79 × 10** ^−^^**43**^	500.76	7.57 × 10^−13^	0.157 (0.0245)		1.66 × 10^−9^	−3.37 × 10^−4^ (4.36 × 10^−4^)	0.440	499.75	7.15 × 10^−13^
FPG	BMI	91	−0.0353 (0.0207)		0.124	771.11	9.35 × 10^−109^	−0.0294 (0.0321)		0.362	−2.57 × 10^−4^ (1.06 × 10^−3^)	0.807	770.6	3.97 × 10^−109^
BMI	T2DM	296	1.06 (0.0462)	2.89 (2.64–3.17)	**2.42 × 10** ^−^^**116**^	1123.25	7.27 × 10^−97^	1.20 (0.113)	3.32 (2.66–4.14)	6.95 × 10^−22^	−2.68 × 10^−3^ (2.00 × 10^−3^)	0.180	1116.39	4.68 × 10^−96^
T2DM	BMI	75	−8.97 × 10^−3^ (0.0130)		0.570	986.49	1.58 × 10^−159^	−0.0655 (0.0242)		**0.0119**	4.59 × 10^−3^ (1.69 × 10^−3^)	8.12 × 10^−3^	895.61	7.58 × 10^−142^
Childhood BMI	FPG	16	0.0626 (0.0162)		**1.71 × 10** ^−^^**4**^	44.03	1.09 × 10^−4^	7.12 × 10^−3^ (0.0684)		0.919	3.95 × 10^−3^ (4.73 × 10^−3^)	0.418	41.94	1.26 × 10^−4^
FPG	Childhood BMI	77	−0.0589 (0.0376)		0.175	89.4	0.140	−0.119 (0.0590)		0.0641	2.42 × 10^−3^ (1.85 × 10^−3^)	0.195	87.41	0.155
Childhood BMI	T2DM	16	0.579 (0.112)	1.78 (1.43–2.22)	**3.38 × 10** ^−^^**7**^	233.95	2.47 × 10^−41^	0.326 (0.469)	1.39 (0.55–3.47)	0.597	0.0181 (0.0326)	0.587	228.9	6.50 × 10^−41^
T2DM	Childhood BMI	62	−0.0139 (0.0168)		0.491	64.38	3.59 × 10^−1^	−0.0621 (0.0315)		0.0641	4.06 × 10^−3^ (2.26 × 10^−3^)	0.0773	61.09	0.436
FPG	T2DM	81	0.983 (0.138)	2.67 (2.04–3.50)	**2.11 × 10** ^−^^**12**^	2056.4	0	0.288 (0.185)	1.33 (0.93–1.92)	0.185	0.0304 (6.13 × 10^−3^)	3.86 × 10^−6^	1567.12	4.32 × 10^−275^
T2DM	FPG	78	0.133 (9.34 × 10^−3^)		9.73 × 10^−46^	374.23	1.29 × 10^−40^	0.175 (0.0175)		**8.63 × 10** ^−^^**15**^	−3.29 × 10^−3^ (1.18 × 10^−3^)	6.66 × 10^−3^	339.45	5.71 × 10^−35^

The primary estimate of effect was taken from the inverse variance weighted method; where there was evidence of horizontal pleiotropy (intercept *P* < 0.05) the MR-Egger estimate was interpreted as the most relevant estimate. Significant *P*-values (corrected using Benjamini–Hochberg correction) are highlighted in bold.

Number of SNPs, number of single-nucleotide polymorphisms used as instrumental variables; CI, confidence interval; *Q*, *Q* statistic; SE, standard error; IGF-1, insulin-like growth factor 1; BMI, body mass index; WHR, waist–hip ratio; T2DM, type 2 diabetes mellitus; FPG, fasting plasma glucose.

Mediation MR indicated that a substantial proportion of the effect of IGF-1 and childhood BMI on CTS risk operates through adult BMI and T2DM; 33% of the effect of IGF-1 on CTS risk is mediated through higher BMI, while 11% of the effect is mediated through T2DM. Moreover, 15% of the effects of adult BMI are mediated via T2DM and 45% of the effects of childhood BMI are mediated via adult BMI (Table 5).

**Table 5:** Mediation MR analyses.

Exposure	Mediator	Proportion (95% CI; %)
IGF-1	BMI	33.0 (20.9–57.8)
IGF-1	T2DM	10.5 (3.78–25.8)
IGF-1	FPG	6.10 (1.66–16.7)
BMI	T2DM	15.1 (10.4–19.9)
BMI	FPG	5.09 (2.85–7.56)
Childhood BMI	BMI	44.9 (35.8–55.2)
Childhood BMI	T2DM	12.8 (6.99–20.3)
Childhood BMI	FPG	3.50 (1.40–6.49)
T2DM	FPG	23.4 (13.7–34.8)

Mediation analysis was performed using the equation presented in Fig. 2.

IGF-1, insulin-like growth factor 1; BMI, body mass index; WHR, waist–hip ratio; T2DM, type 2 diabetes mellitus; FPG, fasting plasma glucose.

## Discussion

This study provides genetic evidence that elevated plasma IGF-1, obesity, and diabetes are causal drivers of CTS. By integrating univariable and multivariable MR, we demonstrate that the effect of IGF-1 on CTS is partially mediated by adiposity and glycaemic dysregulation. These results offer clearer insights into the interplay between growth factor signalling and metabolic health in the pathophysiology of peripheral nerve compression.

### IGF-1 and CTS

The *DIRC3*-associated CTS-protective variant (rs62175241-T) is associated with higher tenosynovium mRNA (*IGFBP5*) expression [8]. Extracellular IGFBP5 sequesters IGF-1. We propose that localized sequestration attenuates IGF-1 signalling within peri-neural tissues, exerting a protective effect against CTS [8]. Our MR findings provide a causal foundation for this hypothesis, demonstrating that genetically instrumented increases in plasma IGF-1 significantly elevate the odds of CTS. This causal estimate (OR 1.09 per 5.69 nmol/l increase) closely aligns with the ORs of 1.04–1.12 reported in observational literature [8, 11]. The consistency between our results and prior observational data reinforces the role of elevated IGF-1 signalling as a key driver of CTS pathogenesis, bridging the gap between systemic IGF-1 levels and local pathology.

After adjusting for height, the MR estimate for IGF-1 on CTS increased, identifying height as a negative confounder. Higher plasma IGF-1 promotes taller stature, which we identify as protective for CTS; height may partially offset the detrimental effects of elevated IGF-1 on CTS risk. This protective influence may be driven by anatomical scale; taller individuals may have larger carpal tunnel dimensions, accommodating higher pressures before median nerve compression. The specific relationship between stature and carpal tunnel volume remains to be fully characterized.

The causal effects of elevated plasma IGF-1 attenuated when adjusted for elevated BMI, WHR, T2DM and FPG, suggesting these metabolic factors partially confound the effect. There is an inverse relationship between plasma IGF-1 and BMI in adults; thus, this result appears paradoxical [31]. However, this likely reflects the complex, time-varying biology of IGF-1 which is not captured by MR’s assumption of a constant lifecourse exposure. Consistent with this interpretation, raised childhood BMI is associated with elevated plasma and urinary IGF-1 [26]. We hypothesize that these findings may reflect a negative feedback loop, whereby elevated plasma IGF-1 initially leads to elevated BMI, which subsequently inhibits further IGF1 production. IGF-1 exhibits antagonistic pleiotropy in diseases including dementia, vascular disease, osteoporosis, generalized mortality, and notably diabetes, in which higher IGF-1 is protective earlier in life but associated with increased risk in older age [55]. Further analyses are required to elucidate sex-specific effects linking adiposity, IGF-1 levels, and risk of CTS.

### Obesity and CTS

Elevated BMI, WHR and WC increased odds of CTS in univariable MR estimates. The effect estimates for BMI and WHR did not attenuate on multivariable MR, implying that central and peripheral adiposity independently increase liability to CTS. These findings are consistent with previous two-sample MR results using different datasets [36, 37], adding further weight to the causal relationship between obesity and CTS. Moreover, we demonstrate that childhood BMI also increases CTS risk, an effect substantially mediated by elevated adult BMI.

Several mechanisms link obesity with CTS. As a chronic, systemic pro-inflammatory state [56], obesity can drive local inflammation, which plays a key role in compression neuropathies [57]. Inflammation can increase the cross-sectional area of nerves [57] which results in nerve compression within confined anatomical spaces such as the carpal tunnel. The pro-inflammatory effects of obesity also increase the severity of neuropathic pain [58], further compounding the impact on nerve health.

### Glycaemic traits and CTS

We identify T2DM as a causal risk factor for CTS, independent of factors associated with obesity, FPG, or plasma IGF-1. We also implicate FPG in CTS, complementing previous findings [36] from different datasets. This adds weight to the hypothesis that raised glucose levels in diabetes cause median nerve damage. Hyperglycaemia increases nerve susceptibility to local factors such as pressure, strain, elongation, and local connective tissue changes involved in median nerve entrapment in the carpal tunnel [59] and may also contribute to nerve swelling by osmosis [60]. However, the effect of FPG on risk of CTS attenuates when adjusted for T2DM, implying that the effects of elevated FPG on CTS risk are not driven by transient elevations in glucose alone, but through the multifaceted phenotype that comprises T2DM.

A ‘two hit’ theory is proposed by which T2DM may influence CTS susceptibility beyond hyperglycaemia alone [61]. Patients with T2DM, but without CTS, have a higher median nerve cross-sectional area—a hallmark of CTS [61]. Microvascular events in the flexor tendon sheath may also increase nerve susceptibility to injury and thus CTS [62], as may oxidative damage due to elevated ATP production [63]. Advanced glycation end-product accumulation in connective tissue surrounding the median nerve could also contribute to CTS pathogenesis [64].

Leave-one-out analysis demonstrated that the causal relationship between T1DM and CTS was due to a single SNP, rs9273364. This SNP resides upstream of the HLA-DQB1 gene, which plays a major role in genetic predisposition to T1DM [65]. We postulate that there may be a shared immune or inflammatory predisposition to T1DM and CTS which warrants further investigation.

### Insights from bidirectional and mediation Mendelian randomization

The inverse relationship between BMI and IGF-1—where higher adult BMI is observationally linked to lower IGF-1, yet CTS patients exhibit elevated IGF-1—presents a metabolic paradox. Our bidirectional MR analyses (Table 4) identify a regulatory feedback loop: while genetically instrumented IGF-1 increases BMI, genetically instrumented BMI conversely lowers plasma IGF-1.

**Figure 5:**
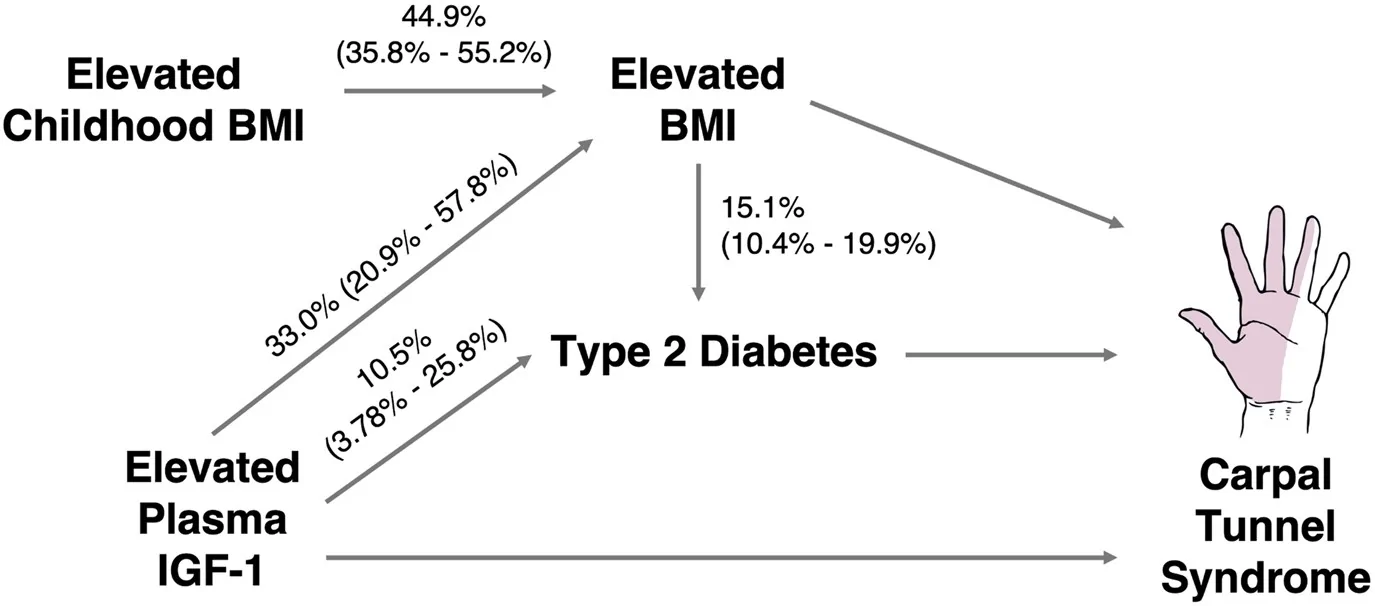
Directed acyclic graph (DAG) summarizing the results from mediation MR analysis (95% confidence intervals shown in brackets).

Furthermore, mediation MR reveals a hierarchical pathway of risk: the effects of IGF-1 on CTS are partially channelled through increased BMI and T2DM risk, while the impact of obesity on CTS is itself partially mediated by T2DM. This suggests that IGF-1 acts both as a direct driver of nerve pathology and an upstream regulator of metabolic dysfunction (Fig. 5).

The divergent causal relationships underscore the potential for antagonistic pleiotropy where the biological role of the IGF-1 axis shifts across the life course or in response to varying metabolic states. Such complexity highlights the necessity of using MR to disentangle lifelong exposures from the cross-sectional ‘snapshots’ provided by traditional observational data.

### Limitations

It would be prudent to replicate analyses in individuals of different ancestral populations as this study leverages exclusively European population data, limiting generalizability. Many IVs are drawn from the UK Biobank, with the potential for overlap between the exposure and outcome populations. The effects of sample overlap in MR may not be as problematic as previously thought [35, 66], particularly in the context of strong IVs such as those in our study (which all showed mean F statistics >10). Finally, the power of MR estimates is limited for exposures where few genetic instruments were available, resulting in substantial heterogeneity in several analyses, although the estimates generated were robustly replicated using weighted median MR and MR-PRESSO (Supplementary Tables S4 and S5), strengthening the evidence that causal inference may be drawn from these estimates.

### Clinical relevance

We implicate general and central adiposity in the pathophysiology of CTS. It remains to be seen whether weight loss ameliorates CTS symptoms in patients with higher BMI or WHR, as is observed in patients undergoing bariatric surgery [23]. There is much interest in incretin mimetic therapies used to treat T2DM and obesity, such as semaglutide and tirzepatide [67] and a study in patients with CTS is warranted. Additionally, we identify hyperglycaemia, T1DM, and T2DM as causal risk factors for CTS. Improved glycaemic control may present a therapeutic opportunity to prevent or improve the symptoms of CTS in individuals with diabetes.

The relationship between higher IGF-1 and CTS represents a promising opportunity for drug repurposing. Many existing drugs currently target IGF-1 signalling, such as metformin, first-line treatment for T2DM, which also downregulates IGF-1 receptor signalling [68], and teprotumumab, a monoclonal antibody used to treat thyroid eye disease [69]. Future studies should evaluate the role of these therapies in CTS.

In summary, using MR, we show that obesity, T2DM, and higher plasma IGF-1 are independent, causal risk factors in the pathophysiology of CTS. Reported associations between T2DM and CTS occur, in part, via increased plasma glucose levels. Finally, we highlight opportunities for drug repurposing and drug-target studies to improve the clinical care provided to patients with CTS.

## Ethics approval

None required.

## Supplementary Material

dyag173_Supplementary_Data

## Data Availability

Not applicable.
