# Supplementary material for: Examining the role of insulin-like growth factor 1 in the pathophysiology of carpal tunnel syndrome: a Mendelian randomization study
Source: Int J Epidemiol. 2026 Aug 27;55(5):dyag173. doi: 10.1093/ije/dyag173 (PMC13520936; doi:10.1093/ije/dyag173)
Supplement: Supplementary file 1 [file dyag173_supplementary_data.zip › ije-2025-09-1823-File009.docx]

***Supplementary Figure 1:*** *Scatter plots showing results for significant exposures from univariable MR.*
